# Supplementary material for: Intracellular calcium leak lowers glucose storage in human muscle, promoting hyperglycemia and diabetes
Source: eLife. 2020 May 4;9:e53999. doi: 10.7554/eLife.53999 (PMC7282812; doi:10.7554/eLife.53999)
Supplement: Supplementary file 1. [file elife-53999-supp1.docx]

### Supplemental File 1. Full Proteomics Report Table

|  | | Unique Peptide Count | | | | | | | | |
| --- | --- | --- | --- | --- | --- | --- | --- | --- | --- | --- |
| Region in Gel (see figure) | |  | A | | | | B | | | |
| Identified Proteins (55/57) OS =Homo sapiens | Accession | M W kDa | 119 MHS | 108 MHS | 117 MHN | 111 MHN | 119 MHS | 108 MHS | 117 MHN | 111 MHN |
| Cluster of Glycogen phosphorylase, muscle form GN=PYGM SV=6 | PYGM_HUMAN [3] | 97 |  |  |  |  | 62 | 68 | 49 | 46 |
| Glycogen phosphorylase, muscle form | PYGM_HUMAN | 97 |  |  |  |  | 59 | 66 | 48 | 45 |
| Glycogen phosphorylase, brain form | PYGB_HUMAN | 97 |  |  |  |  | 16 | 16 | 11 | 10 |
| Glycogen phosphorylase, liver form | PYGL_HUMAN | 97 |  |  |  |  | 11 | 11 |  |  |
| Glycogen debranching enzyme GN=AGL PE=1 SV=3 | GDE_HUMAN | 175 | 23 | 18 | 5 | 10 | 19 | 13 | 1 | 8 |
| SERCA Ca ATPase 1 GN=ATP2A1 PE=1 SV=1 | AT2A1_HUMAN | 110 | 4 | 2 | 1 | 11 | 44 | 41 | 30 | 39 |
| SERCA 2 | AT2A2_HUMAN | 115 | 1 | 1 |  | 7 | 43 | 38 | 22 | 38 |
| Transitional ER ATPase GN=VCP PE=1 SV=4 | TERA_HUMAN | 89 |  |  |  |  | 25 | 18 | 10 | 23 |
| Cluster of Keratin, type I cytoskeletal 10 GN=KRT10 PE=1 SV=6 | K1C10_HUMAN [5] | 59 |  |  |  |  | 1 | 55 | 3 |  |
| Keratin, type II cytoskeletal 1 GN=KRT1 PE=1 SV=6 | K2C1_HUMAN | 66 |  |  | 1 |  | 7 | 28 | 6 | 3 |
| Keratin, type I cytoskeletal 9 GN=KRT9 PE=1 SV=3 | K1C9_HUMAN | 62 |  |  |  |  | 3 | 23 | 1 |  |
| Cluster of Na/K ATPase α-2 GN=ATP1A2 PE=1 SV=1 | AT1A2_HUMAN | 112 |  |  |  |  | 18 | 10 |  | 17 |
| Alpha-actinin-2 GN=ACTN2 PE=1 SV=1 | ACTN2_HUMAN | 104 |  |  |  |  | 4 | 20 | 1 | 16 |
| Glycogen synthase, muscle GN=GYS1 SV=2 | GYS1_HUMAN | 84 |  |  |  |  | 11 | 10 | 2 | 5 |
| Cluster of Keratin, type II cytoskeletal 6A GN=KRT6A PE=1 SV=3 | K2C6A_HUMAN [2] | 60 |  |  |  |  |  | 25 | 1 |  |
| Fructose-bisphosphate aldolase A PE=1 SV=2 | ALDOA_HUMAN | 39 |  |  |  |  | 1 |  | 2 | 5 |
| Q9HC35-DECOY | Q9HC35-DECOY | ? |  |  |  |  | 1 | 1 | 1 | 1 |
| Endoplasmin OS=Homo sapiens GN=HSP90B1 PE=1 SV=1 | ENPL_HUMAN | 92 |  |  |  |  | 5 | 1 |  | 10 |
| Calnexin GN=CANX PE=1 SV=2 | CALX_HUMAN | 68 |  |  |  |  | 5 | 3 |  | 5 |
| Desmoplakin GN=DSP PE=1 SV=3 | DESP_HUMAN | 332 |  |  |  |  |  | 11 |  |  |
| Keratin, type II cytoskeletal 5 GN=KRT5 PE=1 SV=3 | K2C5_HUMAN | 62 |  |  |  |  |  | 17 | 1 |  |
| Protein Wnt-10a GN=WNT10A PE=1 SV=1 | WN10A_HUMN | 46 |  |  |  |  | 1 | 1 |  | 1 |
| 6-phosphofructokinase, muscle type GN=PFKM PE=1 SV=2 | PFKAM_HUMAN | 85 |  |  |  |  | 5 | 1 |  | 3 |
| 26S proteasome non-ATPase regulatory subunit 2 GN=PSMD2 PE=1 SV=3 | PSMD2_HUMAN | 100 |  |  |  |  | 2 | 1 |  | 2 |
| Ryanodine receptor 1 GN=RYR1 PE=1 SV=3 | RYR1_HUMAN | 565 |  |  |  | 2 |  |  |  |  |
| Calsequestrin-1 GN=CASQ1 PE=1 SV=3 | CASQ1_HUMAN | 45 |  |  |  |  | 2 | 2 |  | 2 |
